# First‐time application of droplet digital PCR for methylation testing of the 11p15.5 imprinting regions

**DOI:** 10.1002/mgg3.2264

**Published:** 2023-07-31

**Authors:** Elia Schlaich, Wouter H. G. Hubens, Thomas Eggermann

**Affiliations:** ^1^ Institute for Human Genetics and Genome Medicine, Medical Faculty, RWTH Aachen University Aachen Germany; ^2^ Institute for Stem Cell Biology, Medical Faculty, RWTH Aachen University Aachen Germany; ^3^ Helmholtz Institute for Biomedical Engineering, RWTH Aachen University Aachen Germany

**Keywords:** imprinting disorders, mosaicism, MS‐ddPCR, MS‐MLPA, MS‐pyrosequencing

## Abstract

**Background:**

Beckwith‐Wiedemann syndrome and Silver‐Russel syndrome are two imprinting disorders caused by opposite molecular alterations in 11p15.5. With the current diagnostic tests, their molecular diagnosis is challenging due to molecular heterogeneity and mosaic occurrence of the most frequent alterations. As the determination of precise (epi)genotype of patients is relevant as the basis for a personalized treatment, different approaches are needed to increase the sensitivity of diagnostic testing of imprinting disorders.

**Methods:**

We established methylation‐specific droplet digital PCR approaches (MS‐ddPCR) for the two imprinting centers in 11p15.5, and analyzed patients with paternal uniparental disomy of chromosome 11p15.5 (upd(11)pat) and other imprinting defects in the region. The results were compared to those from MS‐MLPA (multiplex ligation‐dependent probe amplification) and MS‐pyrosequencing.

**Results:**

MS‐ddPCR confirmed the molecular alterations in all patients and the results matched well with MS‐MLPA. The results of MS‐pyrosequencing varied between different runs, whereas MS‐ddPCR results were reproducible.

**Conclusion:**

We show for the first time that MS‐ddPCR is a reliable and easy applicable method for determination of MS‐associated changes in imprinting disorders. It is therefore an additional tool for multimethod diagnostics of imprinting disorders suitable to improve the diagnostic yield.

## Graphical abstract

We describe for the first time the application of droplet digital PCR to identify aberrant methylation in imprinting disorders. As a proof of principle of the diagnostic suitability of the method to detect aberrant epigenetic patterns, we have chosen DNA samples with specific aberrant imprinting patterns. We could show the reliability of the technique by comparing it with two other well established methods (MS‐MLPA, MS‐pyrosequencing).

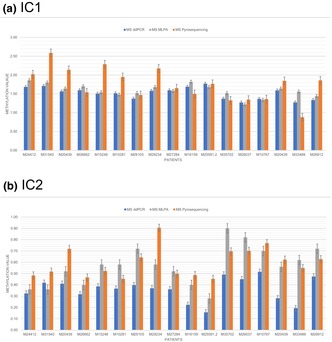

## 1 INTRODUCTION

Beckwith‐Wiedemann syndrome (BWS) and Silver‐Russell syndrome (SRS) belong to the group of congenital imprinting disorders (ImpDis) which are characterized by the altered regulation of imprinted genes. These genes are expressed monoallelically depending on their inheritance either from the mother or from the father (Brioude et al., 2018). Currently, 13 ImpDis have been clinically defined, which are associated with molecular alterations at differentially methylated regions (DMRs) on chromosomes 6, 7, 8, 11, 14, 15, and 20. The phenotypes of ImpDis include aberrant growth, development, and metabolism (Butler, 2020). In some ImpDis, these phenotypes overlap, despite different molecular and (epi)genetic etiologies, whereas for other ImpDis minor molecular alterations can cause completely opposite phenotypes. For instance, both BWS and SRS have DMR alterations on chromosome 11p15.5 but are characterized by overgrowth (BWS) or growth retardation (SRS).

In approximately 80% of patients with BWS, molecular disturbances of the DMRs in 11p15.5 can be observed, comprising imprinting defects (ImpDef) (55%–60%), paternal uniparental disomy of 11p15.5 (upd(11)pat) (20%), and mutations of the *CDKN1C* gene (5%) (Wang et al., 2019). The most common ImpDef are loss of methylation (LOM) at the maternal *KCNQ1OT1*:TSS‐DMR (imprinting center 2, IC2) (50%) and gain of methylation (GOM) at the maternal *H19/IGF2*:IG‐DMR (IC1) (5%–10%); for review: (Brioude et al., 2018). SRS is associated with LOM of the imprinting center 1 (IC1) (30%–60%) or maternal uniparental disomy of chromosome 7 (5%–10%) as the main molecular alterations; for review: (Wakeling et al., 2017).

Clinical features of BWS include macrosomia, hemihypertrophy, macroglossia, and abdominal wall defects; for review: (Brioude et al., 2018). The disorder is also associated with an increased risk for embryonal tumors, in particular Wilms tumor. SRS is clinically characterized by intrauterine and postnatal growth restriction, relative macrocephaly at birth, postnatal growth retardation, protruding forehead, body asymmetry and feeding difficulties, and/or low BMI; for review: (Wakeling et al., 2017).

In BWS, epigenotype–phenotype correlations for some features have been reported (Coktu et al., 2020; Mussa et al., 2016), emphasizing the importance to identify the precise molecular change as it has an impact on clinical management and patient outcome. In particular, the risk of embryonal tumors is lower in BWS patients with IC2 LOM than in IC1 GOM or upd(11)pat. However, routine diagnostic testing can be challenging due to (low‐level) mosaicism for upd(11)pat and ImpDef (Duffy et al., 2019). As a result, these molecular alterations might escape diagnostic detection, particularly as routine testing is commonly restricted to blood samples.

For diagnostic testing of BWS and SRS, several methods are routinely applied, the most common is the commercially available methylation‐specific multiplex ligation‐dependent probe amplification (MS‐MLPA) assay. In fact, it has been discussed to be less sensitive as other techniques for detecting low‐level mosaicism (Baker et al., 2021; Wang et al., 2019). However, several studies indicate that multimethod assays and testing of different tissues are currently the most appropriate approaches to address mosaicism (Baker et al., 2021; Romanelli et al., 2011; Russo et al., 2016), though even these strategies probably miss the detection of low‐level mosaic constitutions.

In order to improve diagnostic testing, we determined the suitability of bisulfite droplet digital PCR (in the following called methylation‐specific/MS‐ddPCR) to detect aberrant imprinting in the two imprinting centers (ICs) in 11p15.5 in general and in cases with different ratios of mosaicisms and compare it to other methods.

MS‐ddPCR is a probe‐based PCR technique in which two fluorescence‐labeled probes are added that target the CpG of interest (Yu et al., 2015). One of these probes binds to the methylated allele, the other to the non‐methylated allele. Thousands of nanoliter‐sized droplets, each containing (optimally) one bisulphite‐converted DNA molecule, are generated prior to PCR. Depending on the allele in a droplet, one of the two fluorescence‐labeled probes binds. After amplification, the fluorescence signal is read for each individual droplet and the ratio between the two fluorescent probes can be calculated, thus revealing the percentage of methylation of the analyzed CpG.

Though MS‐ddPCR has already been applied to analyze methylation patterns in humans (Han et al., 2020; Sontag et al., 2022; Van Wesenbeeck et al., 2018; Zemmour et al., 2018), imprinted loci have only been addressed by non‐MS‐ddPCR approaches so far (Hartin et al., 2019; Romanet et al., 2019). We here describe the use of MS‐ddPCR for the first time to determine aberrant methylation in ImpDis, to assess whether it is a suitable method for the diagnosis of these disorders.

## 2 METHODS

### 2.1 Study cohort and sample preparation

To establish and validate the MS‐ddPCR approaches, 27 patients with molecularly confirmed BWS (Azzi et al., 2014) and SRS (Wakeling et al., 2017), and 10 healthy control samples were included. They had been analyzed by MS‐MLPA before. Among patients were 17 BWS cases with upd(11)pat and three with IC1 GOM and IC2 LOM each, respectively. Four SRS patients diagnosed with IC1 LOM were included as well.

Bisulfite conversion (BSC) of genomic DNA was performed using the Zymo Research EZ DNA Methylation‐Gold Kit (Zymo Research Corp) according to the manufacturer's protocol with 200 ng of DNA from peripheral lymphocytes, eluted in 20 μL elution buffer.

### 2.2 Targeted CpGs within the IC1 and IC2 in 11p15.5

As a basis for comparison of the reliability of the MS‐ddPCR tests with other methods, CpGs within the two germline DMRs in 11p15.5 were analyzed (Monk et al., 2018). For both IC1 and IC2, one target CpG was selected each. These are also targeted by MS probes of the commercially available MS‐MLPA assay (ME030, MRC Holland) (Table 1).

**TABLE 1:** Primers and probes.

Method	Locus	Primers and probes	Primer sequences	Nt position (hg38)	PCR program
Targeted CpG	IC1			chr11:1998318	
	IC2			chr11:2699336	
MS‐ddPCR	IC1	IC1‐F	ATTTTACGTTTTTGGAGAGTAGG	chr11:1998265–1998287	95°C 10 min, (94°C 30 s, 56.3°C 1 min) × 40, 98°C 10 min, 4°C 30 min, 4°C forever
		IC1‐R	AACACAAACTCRATCAACTAAAT	chr11:1998365–1998387	
		IC1‐FAM^a^	AGGGAAGTGTCGTAAATTTTTTGGT	chr11:1998308–1998332	
		IC1‐HEX^a^	AGAGGGAAGTGTTGTAAATTTTTTGGT	chr11:1998306–1998332	
	IC2	IC2‐F	GTTAGGTTGTATTGTTGAYG	chr11:2699271–2699290	95°C 10 min, (94°C 30 s, 52°C 1 min) × 40, 98°C 10 min, 4°C 30 min, 4°C forever
		IC2‐R	CCTCCCCATCTCTCTAA	chr11:2699398–2699414	
		IC2‐FAM^a^	GGGTATATAGTTTATTTTAGTAACGTTA	chr11:2699313–2699340	
		IC2‐HEX^a^	GGGGTATATAGTTTATTTTAGTAATGTTA	chr11:2699312–2699340	
MS‐pyrosequencing^b^	IC1	IC1‐F	GATGGTATAGAGGGTTTTTTTTTGTT	chr11:1998224–1998250	95°C 15 min, (94°C 45 s, 56°C 45 s, 72°C 1 min) × 50, 72°C 10 min, 4°C forever
		IC1‐R^c^	CAACACAAACTCCATCAACTAAATAAAAAT	chr11:1998359–1998388	
		IC1‐Seq	AGGGGTAGAGGGAAG	chr11:1998300–1998314	
	IC2	IC2‐F	GGAGCGTATTGTTTAGGTTAGGTTGTAT	chr11:2699255–2699282	
		IC2‐R^c^	CCTCCCCATCTCTCTAAAAAAATTTAA	chr11:2699388–2699414	
		IC2‐Seq	GGTTAGGTTGTATTGTTG	chr11:2699270–2699287	
MS‐MLPA	IC1	14792‐L16503^d^	GGTGCTGAGGGGCAGAGGGAAGTGCCGCAAACCCCCTGGTGGGCGCGGTGCCAGCCCCCCA	chr11:1998293‐1998353	
	IC2	16654‐L19204^d^	GCGGGGCACACAGCTCACCTCAGCAACGCCAGTGATCACCCGTCCCGCGCCGTCCGC	chr11:2699310‐2699366	

^a^ Primers are 5′ labeled with FAM or HEX and 3′ labeled with BHQ‐1.

^b^ Information on dispensation order and sequence to analyze are available on request.

^c^ Primers are biotinylated at 5′.

^d^ ME030.

#### 2.2.1 MS‐ddPCR

Primers (Biomers) for the two assays were designed using “Bisulfite Primer Seeker” (Zymo Research; https://www.zymoresearch.de/pages/bisulfite‐primer‐seeker) (Table 1). The probes were 5′ tagged with either 6‐carboxfluorescein (FAM) or hexachloro‐fluorescein (HEX). The 3′ ends were labeled with black hole quencher 1 (BHQ1).

For MS‐ddPCR, 1 μL BSC DNA was mixed with 12 μL ddPCR supermix for probes (no dUTP) (Bio‐Rad Laboratories), 1.2 μL of each primer (20 μM) and FAM‐ and HEX‐probes (5 μM) and 6.2 μL RNase free Aqua dest. For each sample, 21 μL of the mix was added into the middle lane of a DG8 gasket for QX200, in the bottom lane 70 μL of ddPCR droplet generation oil for probes was added. Droplet generation was performed with the QX200 droplet generator (Bio‐Rad Laboratories). Droplet suspension (40 μL) was transferred into a 96‐well ddPCR deep‐well plate, heat‐sealed, and placed in a thermal cycler with the melting temperature of 56.3°C for IC1 and 52°C for IC2 (PCR programs are listed in detail in Table 1). Droplets were quantified using a QX200 droplet reader (Bio‐Rad Laboratories). The results were analyzed with the QuantaSoft Analysis Pro software (Bio‐Rad Laboratories).

Methylation indices (MI) were calculated by dividing the FAM‐positive copies/μL by the sum of FAM‐ and HEX‐positive copies/μL.

To reduce the influence of BSCs efficiency, we adjusted the MI to the controls (control‐adjusted MIs) by dividing the MI of each sample by the mean MIs of the controls of the same BSC within the same run.

All controls were analyzed with at least two different BSC in two independent runs each, to evaluate the impact of BSC and reliability of MS‐ddPCR. Every patient sample was analyzed in at least two independent runs. For a better evaluation of variability between MS‐ddPCR runs, eight controls and two patient samples were analyzed with up to four different BSC and a total of up to six MS‐ddPCR runs (Figure S2).

#### 2.2.2 MS‐pyrosequencing

Primers (Biomers) for MS‐pyrosequencing were designed using the PSQ Assay design software (QIAGEN) (Table 1). PCR was performed with PyroMark PCR Kit (QIAGEN) using a protocol based on the manual of the manufacturer (PCR conditions are shown in Table 1). Pyrosequencing was conducted on a PyroMark Q96 ID (QIAGEN) platform. After the run, all samples were analyzed using the Pyro Q‐CpG software (QIAGEN) and the methylation percentage of the CpG of interest for the IC1 and IC2 assays was used as the result. As with MS‐ddPCR, we calculated the control‐adjusted MIs using the methylated percentage of the samples.

#### 2.2.3 MS‐MLPA

MS‐MLPA with the ME030 assay was performed according to the manufacturer's instructions (MRC Holland). The PCR products were separated by automated capillary electrophoresis (AB3500; Applied Biosystems). The results were analyzed using Coffalyser software (MRC Holland).

#### 2.2.4 Statistical analysis

Statistical significance for the difference in methylation levels between the healthy control cohort and upd(11)pat patients for IC1 and IC2 in both MS‐ddPCR and MS pyrosequencing was determined using the Mann–Whitney *U*‐test using a significance level of *p* < 0.05. Other 11p15.5 epigenotypes were used to confirm the suitability of the test but were not statistically analyzed due to the small cohort size.

## 3 RESULTS

As MS‐ddPCR has not yet been used to address aberrant methylation in ImpDis, the suitability of the technique was evaluated by two assays targeting the IC1 and the IC2 in 11p15.5 in samples with known imprinting patterns obtained by MS‐MLPA. The MS‐ddPCR results were compared with other MS methods by determining the methylation levels for the same two CpGs in the IC1 and IC2, respectively (Tables 1 and 2).

**TABLE 2:** Mean control‐adjusted methylation indices for all patients of each epigenotype group analyzed by methylation‐specific droplet digital PCR approaches (MS‐ddPCR) and MS‐pyrosequencing, as well as mean doubled methylation‐specific multiplex ligation‐dependent probe amplification (MS‐MLPA) value.

Epigenotype	MS‐ddPCR	MS‐MLPA	MS‐pyrosequencing
(a) Results for the IC1 DMR in 11p15.5			
Healthy controls	1.00		1.00
upd(11)pat	1.56	1.59	1.81
IC1 LOM	0.22		
IC1 GOM	1.39		
IC2 LOM	0.99		
(b) Results for the IC2 DMR in 11p15.5			
Healthy controls	1.00		1.00
upd(11)pat	0.36	0.57	0.58
IC1 LOM	1.09		
IC1 GOM	0.76		
IC2 LOM	0.12		

### 3.1 MS‐ddPCR

**FIGURE 1:**
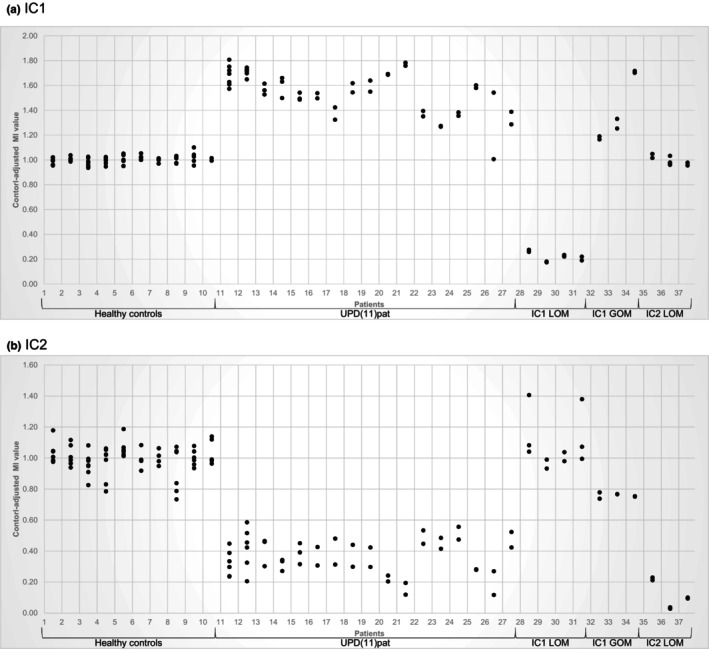
Methylation‐specific droplet digital PCR approaches results for healthy controls, Beckwith‐Wiedemann syndrome (BWS) patients with upd(11)pat and other epigenotypes. The methylation indices (MIs) of the patients were adjusted to the mean MI from at least four healthy control samples within the same run. Each sample was run at least twice. Gain of methylation (GOM) is indicated by values >1, loss of methylation (LOM) by <1. (a) Results for the IC1 differentially methylated regions (DMRs) in 11p15.5. (b) MIs for the IC2 DMR in 11p15.5. (Patients 1–10: healthy controls; patients 11–27: BWS/upd(11)pat of different mosaic degrees; patients 28–31: SRS/IC1 LOM; patients 32–34: BWS/IC1 GOM; patients 35–37: BWS/IC2 LOM).

We confirmed the results obtained from MS‐MLPA in all controls and patients with both IC1 and IC2 assays (Figure 1). The control‐adjusted MIs for the healthy controls varied between 0.94 and 1.10 in the IC1 and between 0.73 and 1.19 in the IC2. The standard deviation (SD) of the MI of the healthy control samples, calculated for each different BSC in each run, varied between 0.01 and 0.02 for the IC1 assay (Figure S1a) and between 0.01 and 0.05 for the IC2 assay (Figure S1b).

To reduce the effect of different BSCs and to assess the reliability of the MS‐ddPCR assays, the MIs of four controls and two upd(11)pat patients from multiple runs were determined. The highest SD of the analyzed samples is 0.03 for IC1 and 0.04 for IC2 (Figure S2).

#### 3.1.1 IC1

Analysis of the BWS patients with upd(11)pat exhibited control‐adjusted MIs commonly ranging from 1.27 and 1.81, corresponding to GOM. These data were significantly different from those of healthy controls (*p* < 0.001). In SRS patients with IC1 LOM, a range of 0.17–0.28 of the control‐adjusted MIs was observed, corresponding to LOM. BWS patients with IC1 GOM revealed control‐adjusted MIs between 1.16 and 1.72. The control‐adjusted MIs of BWS patients with IC2 LOM exhibited a range of 0.96–1.05, corresponding to the healthy control samples (Figure 1a).

#### 3.1.2 IC2

Analysis of the control‐adjusted MIs of upd(11)pat BWS patients resulted in a range of 0.12–0.59 corresponding to LOM. These data differ significantly from those of healthy controls (*p* < 0.001). SRS patients with IC1 LOM had control‐adjusted MIs between 0.93 and 1.41 corresponding to healthy controls. The analysis of the control‐adjusted MIs of the BWS patients with IC1 GOM showed a range between 0.74 and 0.78. This pattern would correspond to a low‐level LOM, but not to the expected result of non‐aberrant methylation. The reason for this observation is currently unclear. Evaluation of the control‐adjusted MIs of patients with BWS having IC2 LOM resulted in a range from 0.03 to 0.23, indicating LOM (Figure 1b).

### 3.2 MS‐pyrosequencing

#### 3.2.1 IC1

Analysis of the BWS patients with upd(11)pat resulted in control‐adjusted MIs between 0.34 and 3.27. In fact, all samples revealed a pattern corresponding to a GOM, with a broad range even in different runs from the same BSC. However, these data were significantly different from those of healthy controls, which ranged from 0.24 to 1.37 (*p* < 0.001). SRS patients with IC1 LOM have control‐adjusted MIs between 0.20 and 0.35, indicating LOM (Figure S3a).

#### 3.2.2 IC2

Determination of the control‐adjusted MIs of patients with upd(11)pat resulted in a range between 0.30 and 1.41. Despite quite significant variation between MS‐pyrosequencing runs, it can be concluded that all samples have LOM, though they showed a considerable variation between the runs (Figure S3b). However, with a *p* value of 0.03, these data were significantly different from those of healthy controls, which ranged from 0.97 to 1.03.

### 3.3 MS‐MLPA

The BWS patients with upd(11)pat exhibited IC1 MIs ranging between 0.61 and 0.93, corresponding to relative GOM hybridization patterns. For IC2, the results varied between 0.14 and 0.45, indicating a relative LOM (Figure S4).

## 4 DISCUSSION

Molecular genetic testing for ImpDis is challenging due to their molecular heterogeneity and the mosaic occurrence of certain molecular subtypes. In routine diagnostics, MS‐MLPA is the most widely applied test to identify aberrant methylation at imprinted loci. However, low‐level mosaicism might escape detection by MS‐MLPA (Baker et al., 2021) and therefore multimethod testing strategies have been proposed to increase the sensitivity of diagnostic testing (Romanelli et al., 2011; Russo et al., 2016). As each of the currently used assays has strengths and limitations for determining aberrant methylation in ImpDis, we aimed to evaluate the suitability of MS‐ddPCR as additional assay for the first time.

Two germline DMRs in 11p15.5 were analyzed in 27 ImpDis patients by MS‐ddPCR and compared to MS‐MLPA. The MS‐ddPCR assays confirmed all MS‐MLPA results, regardless of the underlying molecular alteration, suggesting that MS‐ddPCR can become a promising additional diagnostic tool for ImpDis.

**FIGURE 2:**
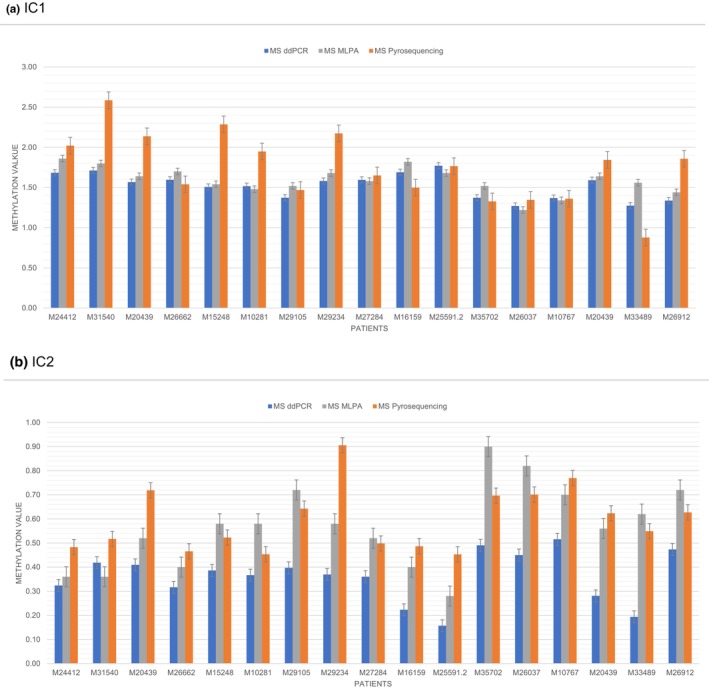
Comparison of methylation testing results for the IC1 (a) and IC2 (b) in 11p15.5 in the upd(11)pat cohort obtained by different MS methods. Mean methylation was calculated based on all results of the same patient in each method. Methylation values of methylation‐specific multiplex ligation‐dependent probe amplification (MS‐MLPA) results are doubled to be comparable with methylation‐specific droplet digital PCR approaches (MS‐ddPCR) and MS‐pyrosequencing results (gain of methylation is indicated by values >1, loss of methylation is indicated by values <1.).

To validate the MS‐ddPCR assay, we chose upd(11)pat patients as the molecular subgroup, because this group shows a wide mosaic range of both relative hypomethylation (IC2) and hypermethylation (IC1) in the same sample, thus providing a representative overview of the suitability of the new assay. The results were consistently reproducible in different BSC and in different MS‐ddPCR runs (Figure 1). Levels of methylation were comparable between MS‐ddPCR and MS‐MLPA. Interestingly, in case of the IC2 LOM we found that MS‐ddPCR exhibited lower methylation values than MS‐MLPA. The reason for this finding is currently unclear, and further studies are needed to confirm this observation. In contrast, MS‐pyrosequencing assays showed a wider range for methylation in comparison MS‐ddPCR and MS‐MLPA (Figure 2a,b), whereas MS‐ddPCR proved to be a more robust method in our laboratory (Figure S2).

In contrast to MS‐MLPA the genomic DNA needs BSC treatment prior to MS‐pyrosequencing and MS‐ddPCR, which might impact the result of the subsequent methylation analyses. However, the results from different BSCs in our approach were nearly consistent across all samples of each BSC (Figure 1a,b), though the BSC failed in single samples. Nevertheless, recurrent analysis of the same sample reveals consistent control‐adjusted MIs in MS‐ddPCR.

Several methods are currently applied to determine aberrant imprinting marks, but in the following we refer to our newly developed MS‐ddPCR approach, MS‐MLPA as the most frequently used diagnostic test, and MS‐pyrosequencing which is also used by several diagnostic laboratories (Mackay et al., 2022).

MS‐MLPA has the advantage of being commercially available and requires common diagnostic laboratory equipment. It allows the identification and differentiation of CNVs, UPDs and ImpDefs in the same run and—as multiplex assay—several DMRs can be targeted at the same time. However, its sensitivity to detect low‐level mosaic constellations has been questioned (Baker et al., 2021; Demars et al., 2010). MS‐ddPCR can only test single CpGs, but the technique is flexible as it comprises customized primers and probes allowing specific CpGs to be targeted. Methylation is quantified absolutely as each droplet is analyzed independently, which reduces PCR bias (Han et al., 2020). Compared with MS‐pyrosequencing, in our laboratory it seems that DNA methylation measurements using MS‐ddPCR are more accurate and may be able to detect lower levels of methylation aberrations than MS‐MLPA. However, its limitations comprise the need of a specific ddPCR equipment which is not available in each diagnostic laboratory, and the additional step of BSC treatment. Finally, the technique detects molecular changes at a specific DMR in general but does not discriminate between the different subtypes (i.e., UPD, CNV, ImpDef). MS‐pyrosequencing has been shown to detect low‐level mosaicism in other studies (Russo et al., 2016), but in our experience it appeared to be less reproducible though we tested several in‐house assays and assays from the literature (Cubellis et al., 2020; Russo et al., 2016).

Recently, the first targeted MS next generation sequencing assay has been published by Ochoa et al. (2021), this assay addresses 63 DMRs including all clinically relevant loci. The primary aim of this approach as well as of other genome‐wide MS assays (e.g., whole genome bisulfite sequencing assays, DNA methylation assays) is to cover a large number of DMRs in order to obtain a comprehensive overview on aberrant methylation of whole genome, but their suitability to detect low‐mosaicism remains to be elucidated.

Thus, it is currently unclear whether deep‐sequencing approaches will improve the detection of low‐level mosaicism. Instead, testing of additional tissues appears to remain the appropriate tool to address mosaicism in patients with a strong suspicion for ImpDis but negative testing results in blood (Azzi et al., 2014; Brioude et al., 2018; Cubellis et al., 2020; Russo et al., 2016; Wakeling et al., 2017).

In conclusion, our data show that MS‐ddPCR is a suitable method for the reliable determination of aberrant methylation in patients with ImpDis. It is rapid and easy to perform and can therefore be used to obtain absolute quantification methylation data from any CpG of interest.

## AUTHOR CONTRIBUTIONS

Elia Schlaich conducted the assays, and Wouter H. G. Hubens supported the data interpretation. Thomas Eggermann was responsible for patient recruitment and the study. Elia Schlaich had drafted the paper, Thomas Eggermann and Wouter H. G. Hubens have commented it.

## FUNDING INFORMATION

The authors are supported by the Deutsche Forschungsgemeinschaft (EG 115/13–1).

## CONFLICT OF INTEREST STATEMENT

The authors declare no conflict of interest.

## ETHICS STATEMENT

The study was approved by the Ethical Committee of the Medical Faculty, RWTH Aachen (EK303‐18).

## PATIENT CONSENT STATEMENT

All participating patients gave informed consent.

## Supporting information


Figure S1.
Figure S2.
Figure S3.
Figure S4.

## Data Availability

Data are available on request.
