# Supplementary material for: First‐time application of droplet digital PCR for methylation testing of the 11p15.5 imprinting regions
Source: Mol Genet Genomic Med. 2023 Jul 31;11(12):e2264. doi: 10.1002/mgg3.2264 (PMC10724498; doi:10.1002/mgg3.2264)
Supplement: Supplementary file 1 — Figure S1. Figure S2. Figure S3. Figure S4. [file MGG3-11-e2264-s001.pptx]

## Slide 1
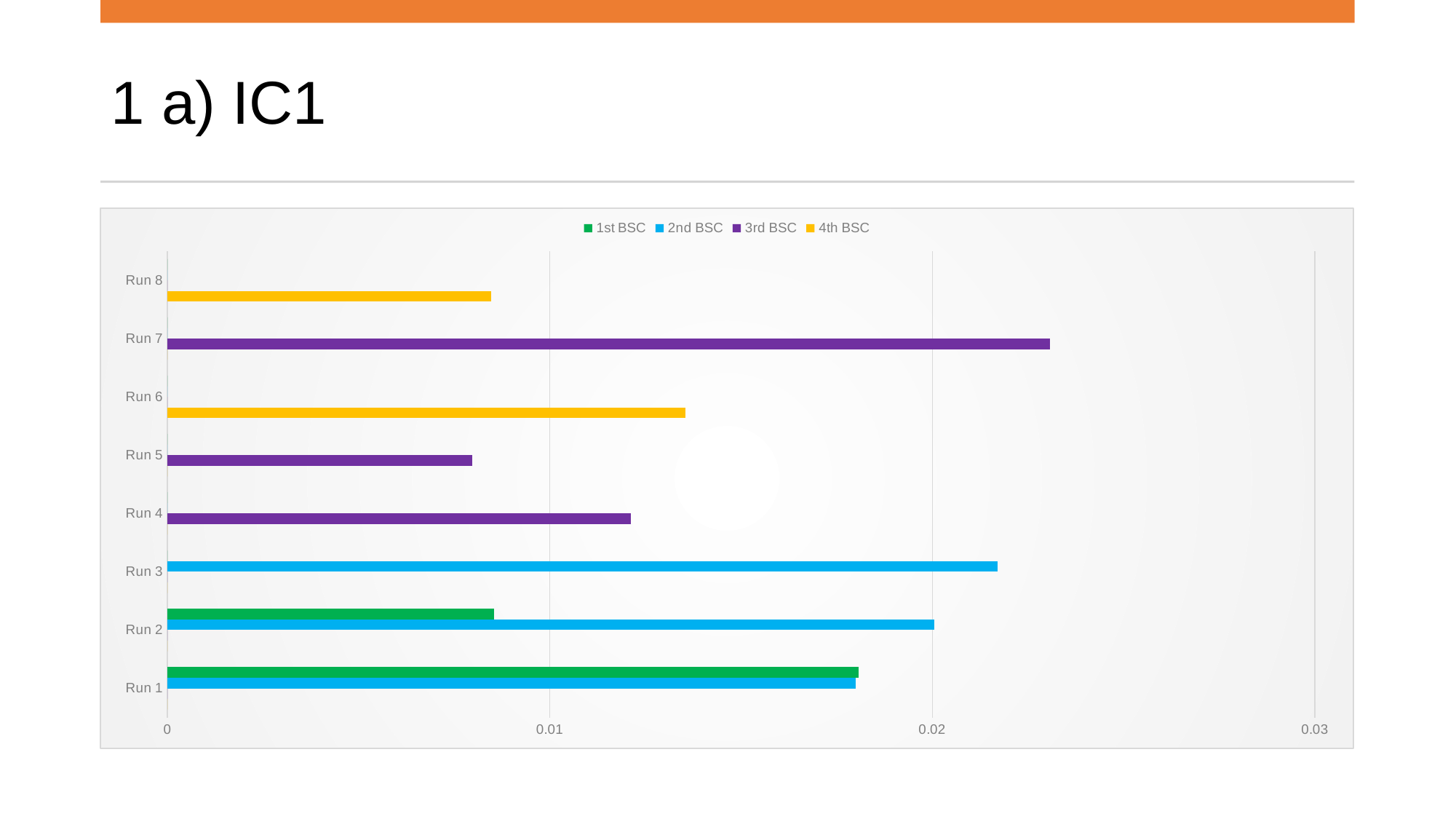

# 1 a) IC1
### Chart
| Category | | | | |
|---|---|---|---|---|
| Run 1 | None | None | 0.017996125477390046 | 0.018068479260351704 |
| Run 2 | None | None | 0.02004888787989191 | 0.008534067179237012 |
| Run 3 | None | None | 0.02170197150126913 | None |
| Run 4 | None | 0.012125727077339195 | None | None |
| Run 5 | None | 0.007979599963291333 | None | None |
| Run 6 | 0.013553527487486887 | None | None | None |
| Run 7 | None | 0.02306701973578029 | None | None |
| Run 8 | 0.0084550847097787 | None | None | None |

## Slide 2
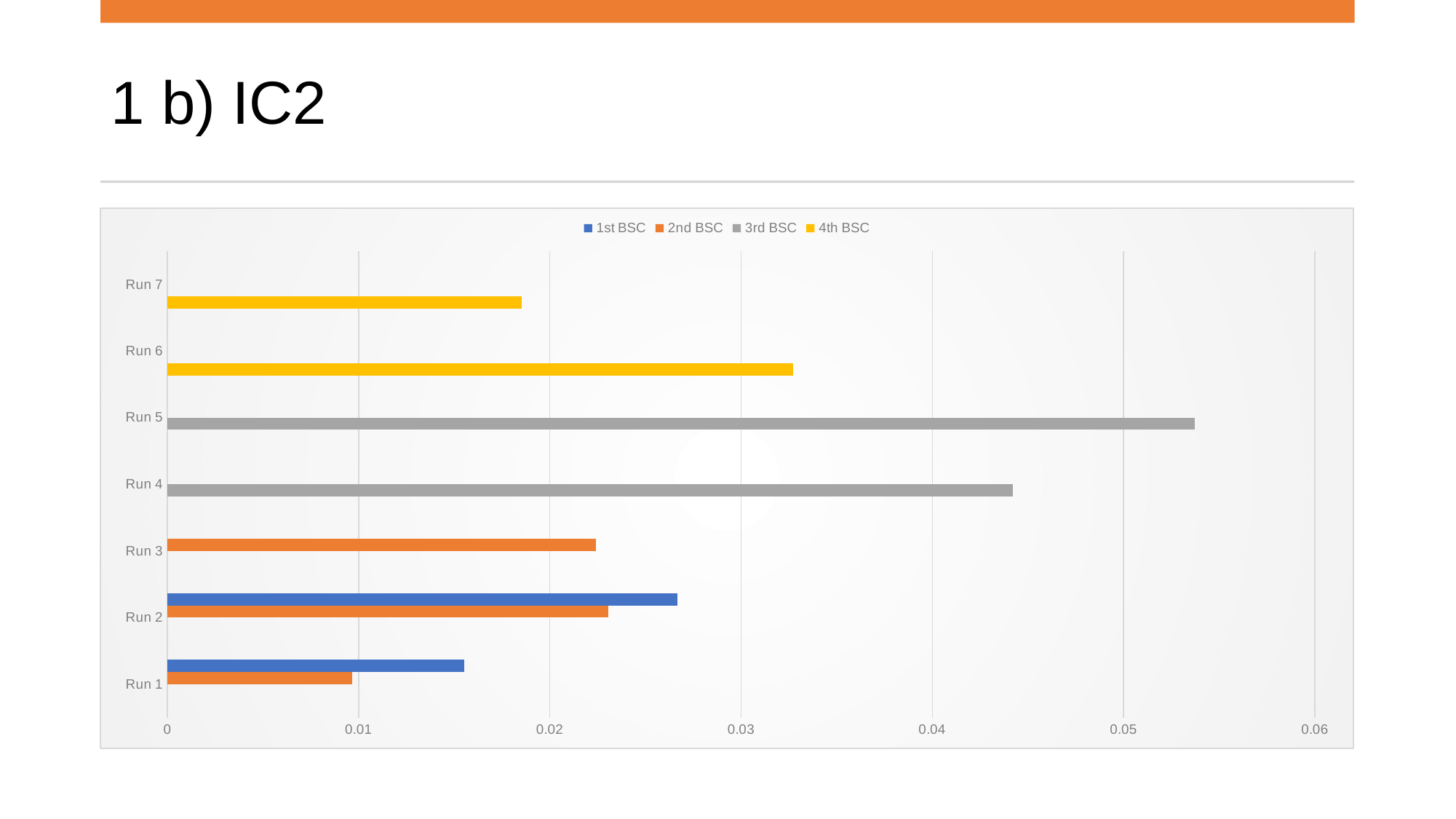

# 1 b) IC2
### Chart
| Category | | | | |
|---|---|---|---|---|
| Run 1 | None | None | 0.009649616478838089 | 0.015515924193089487 |
| Run 2 | None | None | 0.023064509130403414 | 0.02666220006554336 |
| Run 3 | None | None | 0.022421032660105023 | None |
| Run 4 | None | 0.044228729925819714 | None | None |
| Run 5 | None | 0.05372914333714341 | None | None |
| Run 6 | 0.03270936116940589 | None | None | None |
| Run 7 | 0.018526507661406854 | None | None | None |

## Slide 3
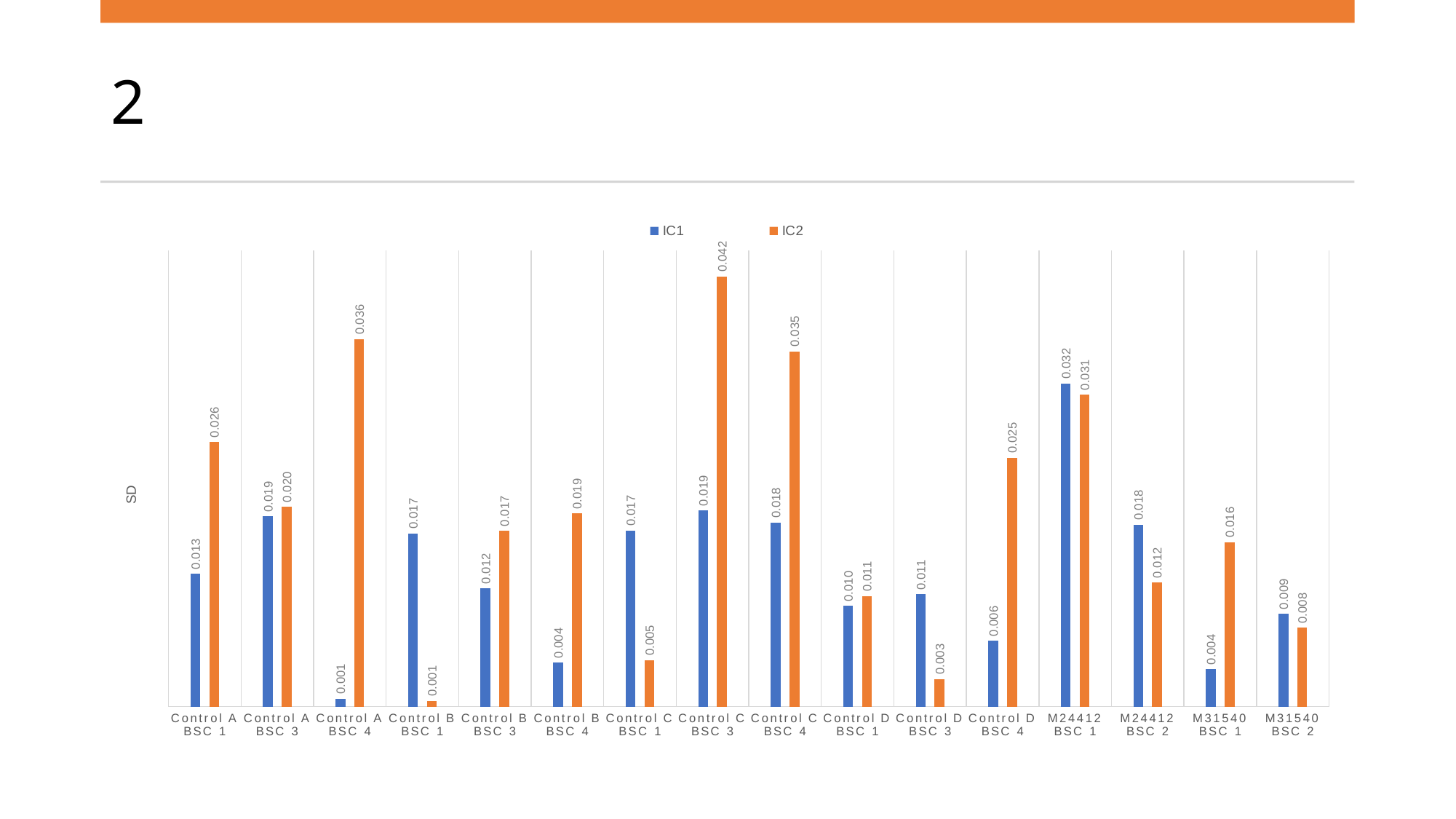

# 2
### Chart
| Category | | |
|---|---|---|
| Control A BSC 1 | 0.013092719879176064 | 0.026121096161094585 |
| Control A BSC 3 | 0.018765475537726853 | 0.019723370498488945 |
| Control A BSC 4 | 0.000769682824885091 | 0.03626448261570092 |
| Control B BSC 1 | 0.017073176267610856 | 0.00056135372384181 |
| Control B BSC 3 | 0.011675297745708807 | 0.017351171696997104 |
| Control B BSC 4 | 0.004321852077357418 | 0.019057368098853708 |
| Control C BSC 1 | 0.017384335832968462 | 0.004579207594676409 |
| Control C BSC 3 | 0.019360048671277903 | 0.04246785337268337 |
| Control C BSC 4 | 0.018160559326197114 | 0.03507243041213897 |
| Control D BSC 1 | 0.009926804865605274 | 0.010897695629241597 |
| Control D BSC 3 | 0.011076462640885298 | 0.002706779271053106 |
| Control D BSC 4 | 0.006488223102889347 | 0.024560240498063962 |
| M24412 BSC 1 | 0.031904743751633574 | 0.03079747331042985 |
| M24412 BSC 2 | 0.01792956265241832 | 0.012240479596966558 |
| M31540 BSC 1 | 0.003677788013338608 | 0.016219601831443844 |
| M31540 BSC 2 | 0.009145393552438607 | 0.00777756068239897 |

## Slide 4
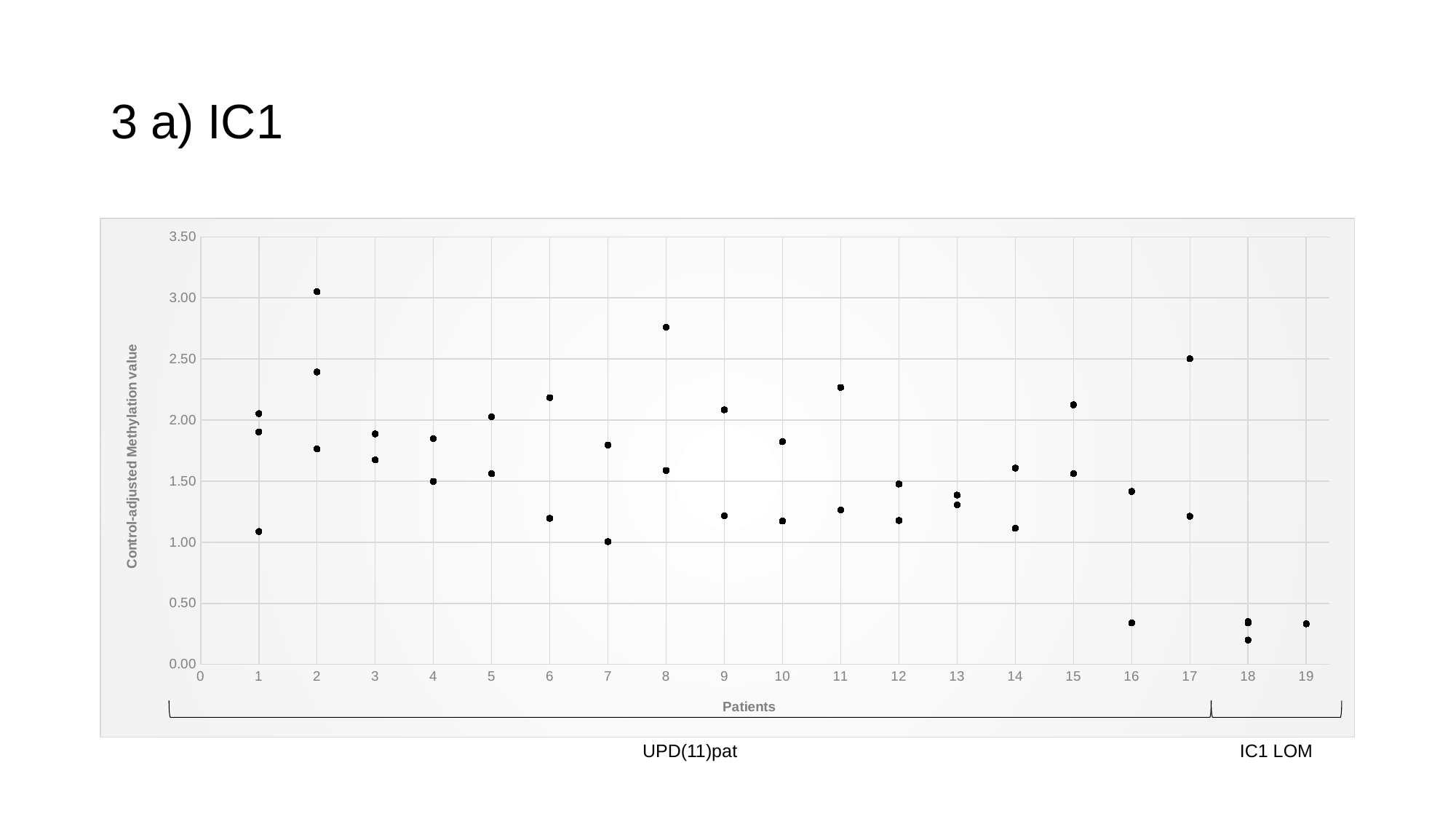

# 3 a) IC1
UPD(11)pat
### Chart
| Category | | | | |
|---|---|---|---|---|IC1 LOM

## Slide 5
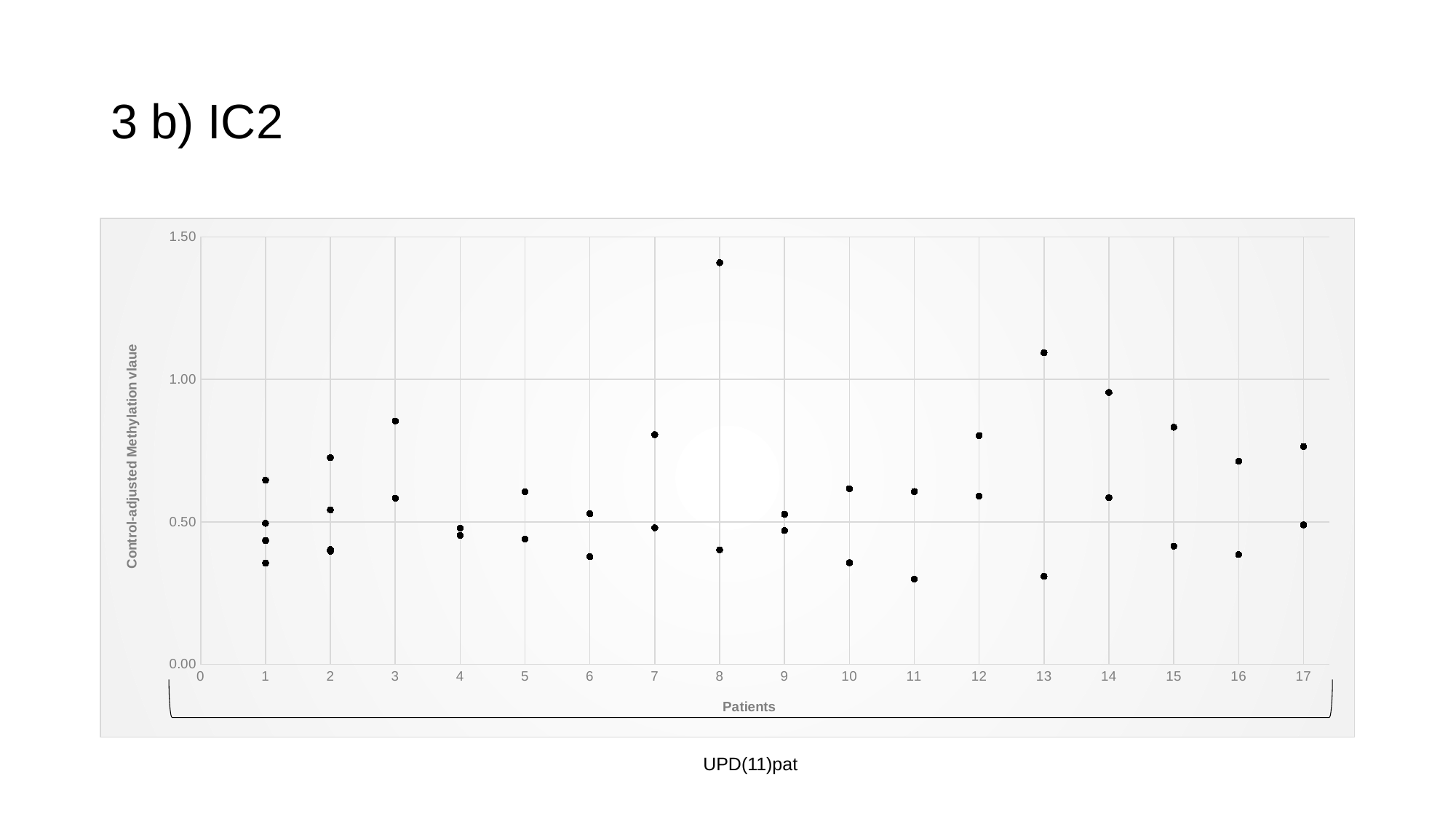

# 3 b) IC2
UPD(11)pat
### Chart
| Category | | | | |
|---|---|---|---|---|

## Slide 6
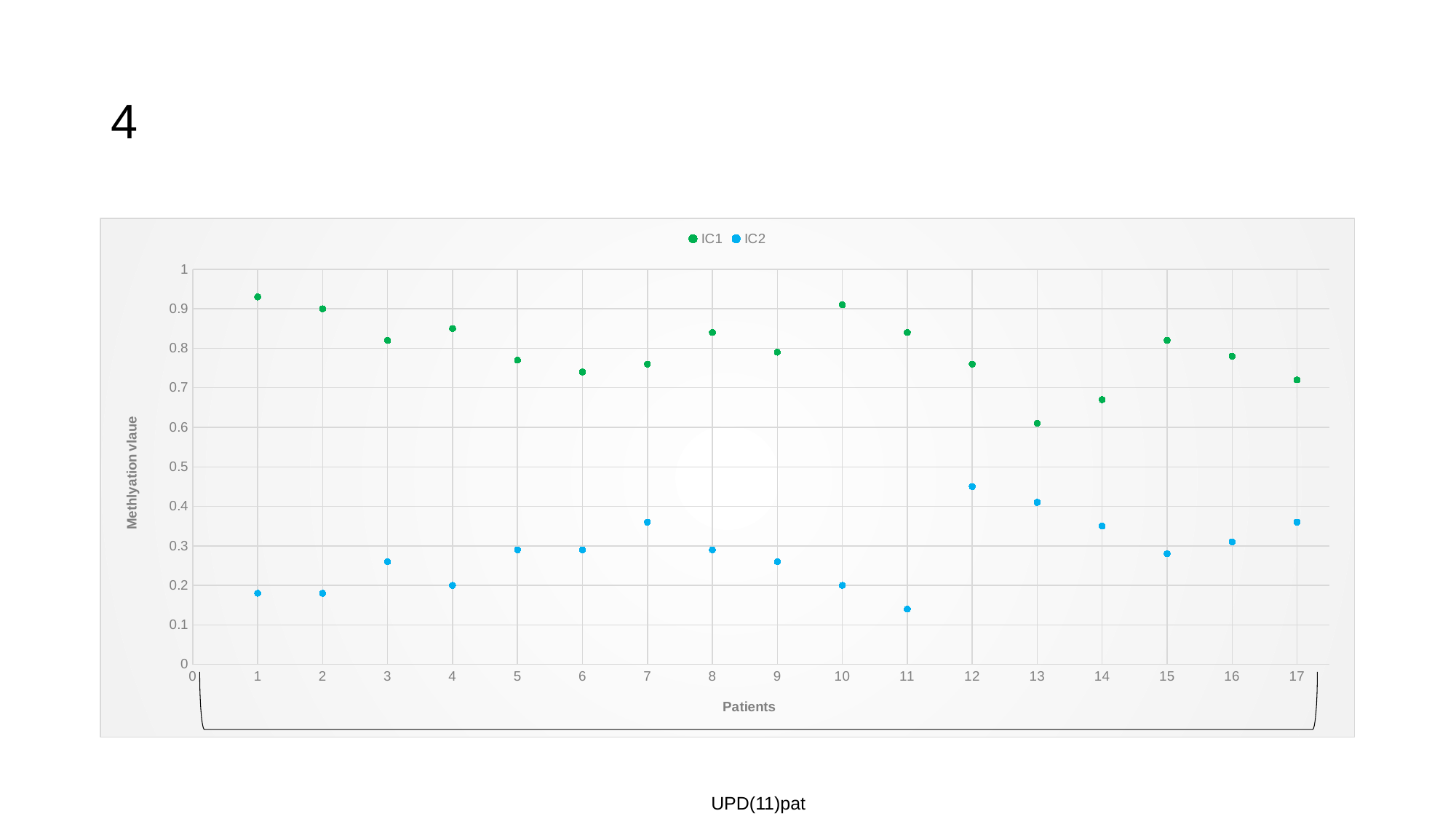

# 4
UPD(11)pat
### Chart
| Category | | |
|---|---|---|
